# Symptomatology of Peripheral Neuropathy in an African Language

**DOI:** 10.1371/journal.pone.0063986

**Published:** 2013-05-15

**Authors:** Asma Shaikh, Alison Bentley, Peter R. Kamerman

**Affiliations:** Brain Function Research Group, School of Physiology, Faculty of Health Sciences, University of the Witwatersrand, Johannesburg, South Africa; "Mario Negri" Institute for Pharmacological Research, Italy

## Abstract

The terminology used to describe neuropathic pain appears to be conserved across languages, which facilitates the translation of validated neuropathic pain screening tools into other languages. However, this assumption has not been assessed in an African language. Therefore we investigated the terminology used by 54 patients whose native language was isiZulu, a major Bantu language of Africa, when describing their symptomatic HIV-associated sensory neuropathy. Also, because English is a commonly spoken second-language in the region, we assessed these patients’ knowledge and understanding of 21 English terms commonly used to describe neuropathic pain. English translations of the most commonly used isiZulu symptom descriptors included: “hot/burning” (50%), “cramping” (35%), “painful/sore/aching” (32%), “itching” (22%), “numb” (22%), “cold/freezing” (17%), and “stabbing/pricking/pins-and-needles” (13%). Thus, the isiZulu terminology to describe neuropathic pain was very similar to that used in non-African languages. However, knowledge and understanding of English neuropathic pain descriptors by these non-native English speakers was highly variable. For example, knowledge of English terms ranged from>98% (“hot”, “cold/freezing”, “cramping”) to <25% (“pricking”, “radiating”, “throbbing”), and true understanding of English terms ranged from>90% (“hot”, “burning”, “cramping”) to <35% (“tingling”, “jumping”, “shooting”, “radiating”). In conclusion, we show significant similarity in the terms used to describe neuropathic pain in isiZulu compared to non-African languages, thus indicating that translation of existing neuropathic pain screening tools into this, and possibly other Bantu languages, is a viable option. However, the usefulness of English-language screening tools in this non-native English speaking population may be limited.

## Introduction

Data on the prevalence and impact of pain of neuropathic origin in Africa are poor. Collecting reliable epidemiological data on neuropathic pain requires an accurate diagnosis, and several neuropathic pain screening tools, which incorporate various combinations of symptoms and clinical signs, have been developed [1], [2]. None of the tools were developed in an African language, so investigators working in Africa either must develop new tools for their study population, translate the semantic components of existing tools into local languages, or use existing tools in the language of origin.

Developing new tools that are tailored to local languages and culture is the optimal approach, but the process is complicated and time consuming, especially in countries with multiple languages (e.g., South Africa has 11 official languages). Instead, translating existing tools into local languages has been the strategy used most commonly outside of Sub-Saharan Africa. In support of this translation approach, numerous studies have demonstrated that the core symptomatology assessed by screening tools remains largely unaltered after translation and validation across diverse language groups [3], [4], [5], [6]. However, although some adjectives used in the semantic component of neuropathic pain screening tools may occur across languages (e.g., “burning”), this may not be true for all the adjectives. For example, the term “tingling” was difficult to translate into Thai [6]. It also is presumptuous to assume that particular terms form part of the language of pain in a novel cultural and language setting [7], [8]. Thus, the translated questionnaire may have face validity, but the content validity may be lower than the original because of cross-cultural differences in the language of pain. An alternative approach is to use screening tools in their original language. This option is viable in Africa because languages such as French and English are frequently spoken as a second or third language. However, the subtleties of abstract pain descriptors such as “pins-and-needles”, “tingling”, and “shooting”, may be lost on non-native speakers of a language, thus reducing the sensitivity and specificity of the tool.

Thus, there are potential problems associated with cross-cultural translation of symptoms and using neuropathic pain screening tools in their original language form in populations who do not speak the language as a first language. Therefore, we investigated the symptomatology of peripheral neuropathy, as described spontaneously by black African patients in their home-language. We also investigated these patients’ understanding of English terms commonly found in neuropathic pain screening tools. We investigated these questions in patients who spoke isiZulu as their home-language, the Bantu language with the second-most native speakers in Africa [9], and who had symptomatic HIV-associated sensory neuropathy, a common cause of peripheral neuropathy in the region [10], [11].

## Materials and Methods

### Ethics statement

The study was approved by the Human Research Ethics Committee (Medical) of the University of the Witwatersrand (clearance number: M090669), and written informed consent was obtained from all participants. An interpreter fluent in isiZulu facilitated the recruitment, consent and interview procedures.

### Participants

Participants were recruited from the Virology Clinic at Charlotte Maxeke Johannesburg Academic Hospital, Johannesburg, South Africa. They were invited to take part in the study if they had a confirmed HIV diagnosis, experienced any pain or abnormal sensations in their feet and lower legs, had signs indicative of a peripheral neuropathy, were on stable antiretroviral therapy for at least one month, and spoke isiZulu as their primary language and English as a secondary language. We defined primary language as the participant’s preferred language of communication when conversing with family and friends. Fifty-four (54) participants met the inclusion criteria and agreed to participate in the interview component of the study.

### Screening for peripheral neuropathy

The ACTG Brief Peripheral Neurological Screening Tool was used to diagnose the presence of HIV-SN [12]. When using the tool in its original form, a positive diagnosis requires the bilateral presence of at least one symptom (burning, aching, pins-and-needles or numbness) and one sign (reduced vibration sense in the great toes or absent ankle reflexes). To avoid any inadvertent priming of participants with English-language descriptors of neuropathic pain during the screening process, we only asked whether the individual had “pain” or “abnormal/odd feelings” in their feet and lower legs. If participants had pain, they rated the intensity of their pain as mild, moderate or severe. No changes were made to the screening tool for the assessment of signs of neuropathy.

### The interview

The interview consisted of two components: firstly, participants were asked to spontaneously describe, in isiZulu, the pain and abnormal sensations in their feet and lower legs, and what triggered the sensations. Patients were not prompted, but were encouraged to give a complete description of all the sensations and triggers for the sensations. Secondly, a list of English neuropathic symptom descriptors was read to participants and they were asked to identify any terms which accurately described their symptoms. Before the list was read to them, it was clarified to the participants that the descriptive words about to be read out referred to the sensations in their lower legs and feet only. Participants were required to answer “yes, I have the symptom”, “no, I do not have the symptom” or “I do not know the term” after each term was read to them. When participants identified themselves as having a symptom, they were asked to provide an equivalent isiZulu term for the English term they had identified. Each interview was recorded and lasted 20 to 30 minutes.

### Development of the English wordlist of neuropathic symptom descriptors

The inventory of English terms used in the list of neuropathic symptoms descriptors was constructed from terms currently used in the English-language versions of the McGill Pain Questionnaire (MPQ) [13], Neuropathic Pain Scale (NPS) [14], Leeds Assessments of Neuropathic Symptoms and Signs (LANSS) [15], Neuropathic Pain Questionnaire (NPQ) [16], Neuropathic Pain Symptom Inventory (NPSI) [17], Douleur Neuropathique en 4 Questions (DN4) [18], and ID Pain [19].

The wordlist initially consisted of 33 words and phrases, but was reduced to 22 items after three pilot interviews showed that none of the patients knew the meaning of the following terms: “bursting”, “strange/unusual”, “squeezing”, “dull”, “intense”, “lancinating”, “gnawing”, “splitting”, “piercing”, “tender” and “sensitive”. This lack of knowledge caused the participants to become visibly embarrassed and uncomfortable during the interview. None of the terms omitted are used in the DN4, LANSS, or ID Pain (tools designed to help identify pain of neuropathic origin). The terms “cold” and “freezing” were combined after the first 3 interviews because participants could not distinguish between the two words, leaving a final list of 21 items (Table 1).

**Table 1:** List of English pain descriptors read to participants.

Final 21-word list of terms	Terms excluded from the original 32-word list after the pilot interviews
Electric/electric shock-like	Bursting
Knife-like/stabbing	Strange/unusual
Pins-and-needles	Squeezing
Tight	Dull
Hot	Intense
Burning	Lancinating
Cold/freezing	Gnawing
Tingling	Splitting
Pricking	Piercing
Jumping	Tender
Shooting	Sensitive
Numb	
Itching	
Caused by heat	
Caused by cold	
Caused by pressure	
Aching	
Throbbing	
Cramping	
Sharp	
Radiating	

### Data analysis

All audio recordings were transcribed and all isiZulu sections of the transcriptions translated into English by two independent translators. In all cases, the two independent translations were compared, and where the translations differed both translators were consulted and a consensus translation agreed on. Data was collected until data saturation was achieved. Descriptive statistics and content analysis were used to analyse the data. We extracted all the terms and phrases used by participants to describe their symptoms and symptom triggers from the original isiZulu transcript and English translation. From the list of English terms read to participants, we calculated the percentage of participants who identified that they had the symptom and the percentage that declared that they did not know the English term.

To confirm whether participants correctly understood the words chosen from the English list we compared the meaning of the English terms chosen to the meaning of the isiZulu terms they provided for each English term. If the back-translation of the isiZulu term matched the English term, or matched an English word belonging to the same grouping of words on the MPQ, then the participant was judged as having understood the English term. For the term “numbness” participants were judged to have this sensation if they described a lack of sensation in their feet and lower legs. Finally a percentage concordance was calculated between the terms and phrases provided during the spontaneous description of neuropathic pain and those selected from the prompted English list to determine whether participants consistently used the same words when describing their neuropathic pain symptoms in both languages.

## Results

Demographic information for the 54 participants is provided in Table 2.

**Table 2:** Demographic and disease-related characteristics of participants.

Characteristic (n = 54, unless otherwise specified)	Mean/Median/Percentage
Mean (SD) age in years	42 (10)
% female	70
Median (range) CD4 T-cell count (cells/mm^3^) (n = 43)^a^	362 (35–837)
Median (range) number of years of formal education (n = 50)^b^	10 (8–12)
Neuropathy signs and symptoms (%)	
Participants with absent ankle reflexes	98
Participants with reduced vibration sense	70
Participants with abnormal sensation (paraesthesias/numbness)	6
Participants with pain	94
Pain intensity in participants reporting pain (%)	
Severe	35
Moderate	37
Mild	28
Antiretroviral exposure (%)	
Stavudine exposure ever	92
Current stavudine use	24
Co-morbidities (%)^a^	
Tuberculosis (n = 51)	37
Alcoholism (n = 51)	10
Diabetes (n = 51)	8

a: Sample <54 because data missing from medical records.

b: Four participants would not divulge their educational level.

### Spontaneous description of neuropathic symptoms in isiZulu

Initial parsing of the spontaneous verbal descriptions showed that the same isiZulu term (*root –*shisa) was used to describe “hot” and “burning” sensation, with the difference in intensity given by the context in which the word was used. Similarly, the contextual use of a single term (*root –hlaba*) was used to indicate the intensity of punctate mechanical sensations “knife-like/stabbing,” “pricking” and “pins-and-needles”. Thus we collapsed the terms describing noxious heat sensations under the heading “hot/burning”, and the terms describing punctate mechanical sensations under the heading “stabbing/pricking/pins-and-needles” for our analyses.

Table 3 shows the English translations of isiZulu terms spontaneously used by participants to describe the quality of their symptoms and what triggered these sensations. Over half of participants used the isiZulu term for “hot/burning” to describe their pain, and about one-third of participants described their pain using the isiZulu terms for “cramping,” or “painful/sore/aching”. Approximately one-fifth of participants said they experienced “itching” or “numbness”. The most common spontaneously divulged trigger for pain was “walking”. Terms equivalent in meaning to “electric-shock”, “sharp”, “throbbing”, “tingling”, “radiating”, “jumping” and “shooting” were not used by any participant when spontaneously describing their neuropathic symptoms in isiZulu.

**Table 3:** English translation of isiZulu terms (in parentheses) spontaneously given by participants to describe their neuropathy symptoms.

Descriptions used by participants (*listed in descending order of frequency*)	Percentage of participants (%) [95% CI]
***Symptom descriptors*** *	
Hot/burning (*root –shisa*; ziyashisa, ukushisa, zinokushisa)	50 [37 to 63]
Cramping (amajaqamba, namacramps, ama-cramps)	35 [29 to 49]
Painful/sore/aching (*root –buhlungu*; zibabuhlungu)	32 [21 to 45]
Itching (*root –luma*; kuyaluma, ziyaluma, ukuluma)	22 [13 to 35]
Numb (ndikindki)	22 [13 to 35]
Cold/freezing (*root –banda*; kubanda, ziyabanda)	17 [9 to 29]
Stabbing/Pricking/Pins-and-needles (*root –hlaba*; ezihlabaya, ziyahlaba, kuyahlaba)	13 [6 to 24]
***Symptom elicitors***	
Pain caused by walking	30 [19 to 42]
Pain caused by pressure/ touch	17 [9 to 29]
Pain caused by cold	4 [1 to 13]
Pain caused by heat	2 [0.3 to 10]

* Where applicable the isiZulu descriptors are listed as the root word followed by adjectival concords commonly used by participants.

### English-language description of neuropathic symptom by isiZulu speakers and understanding of English terms chosen

Table 4 shows the percentage of patients who identified that they had a symptom when they were read a list of English neuropathic pain descriptors, or declared that they did not know the English term. The two most common English symptom descriptors chosen by participants were, “cramping” and “hot”, which were both identified as a symptom by over 85% of participants. However, overall lack of knowledge of English terms was high, with 12 out of the 21 terms (57%) being unknown to more than 20% of participants, and more than 50% of participants did not know the English terms, “radiating”, “stabbing”, “pricking”, “tingling”, “throbbing”, “aching”, and “numbness”.

**Table 4:** Identification and knowledge of English neuropathic symptoms.

Symptom group	Descriptor	Participants who identified that they had the symptom (%) [95% CI]	Participants who did not know the English term (%) [95% CI]
Thermal	Hot	**87 [76 to 94]**	2 [0.3 to 10]
	Burning	**65 [51 to 76]**	11 [5 to 22]
	Cold/freezing	**56 [42 to 68]**	2 [0.3 to 10]
Spatial	Jumping	26 [16 to 39]	24 [15 to 37]
	Shooting	6 [2 to 15]	31 [21 to 45]
	Radiating	7 [3 to 18]	**83 [71 to 91]**
Punctate pressure	Knife-like/stabbing	35 [24 to 49]	**54 [41 to 66]**
	Pricking	9 [4 to 20]	**72 [59 to 82]**
Brightness	Tingling	17 [9 to 29]	**78 [65 to 87]**
	Itching	**59 [46 to 71]**	15 [8 to 23]
Evoked pain	Caused by heat	32 [21 to 45]	2 [0.3 to 10]
	Caused by cold	46 [34 to 59]	2 [0.3 to 10]
	Caused by pressure	**54 [41 to 66]**	6 [2 to 15]
Miscellaneous	Electric-shock	**52 [39 to 65]**	24 [15 to 37]
	Pins-and-needles	43 [30 to 56]	37 [25 to 50]
Incisive pressure	Sharp	39 [27 to 52]	33 [22 to 47]
Temporal	Throbbing	2 [0.3 to 10]	**96 [87 to 99]**
Constrictive pressure	Cramping	**89 [78 to 95]**	2 [0.3 to 10]
	Tight	**61 [48 to 73]**	19 [10 to 31]
Dullness	Aching	13 [6 to 24]	**70 [57 to 81]**
	Numb	30 [19 to 43]	**63 [50 to 75]**

Bolded text shows rates of ≥50%.

Table 5 shows the level of understanding of the 21 English language descriptors by those participants who identified an English word as describing their symptoms. The majority of participants had a true understanding of the thermal descriptors “hot/burning” and “cold/freezing”. So too were the terms, “itching”, “caused by pressure”, “pins-and-needles”, “cramping”, “aching”, and “numb”, well understood, with all six terms being correctly understood by more than 70% of participants who selected the words. The least correctly understood English terms (≤30% correct understanding) were related to descriptions of the spatial characteristics of the pain (“radiating”, “jumping, “shooting”) and the term “tingling”.

**Table 5:** Level of understanding of English-language neuropathic symptoms.

Symptom Group	Descriptor	Number of participants who identified that they had the symptom	Percentage of participants who had a true understanding of the term [95% CI]
Thermal	Hot	47	**98 [89 to 100]**
	Burning	35	**91 [78 to 97]**
	Cold/freezing	30	**83 [66 to 93]**
Spatial	Jumping	14	29 [12 to 55]
	Shooting	3	33 [6 to 79]
	Radiating	4	25 [5 to 70]
Punctate pressure	Knife-like/stabbing	19	58 [36 to 77]
	Pricking	5	60 [23 to 88]
Brightness	Tingling	9	33 [12 to 65]
	Itching	32	**81 [65 to 91]**
Evoked Pain	Caused by heat	17	65 [41 to 83]
	Caused by cold	25	56 [37 to 73]
	Caused by pressure	29	**76 [58 to 88]**
Miscellaneous	Electric-shock	28	61 [42 to 76]
	Pins-and-needles	23	**74 [54 to 87]**
Incisive pressure	Sharp	21	67 [45 to 83]
Temporal	Throbbing	1	100
Constrictive pressure	Cramping	48	**96 [86 to 99]**
	Tight	33	58 [41 to 73]
Dullness	Aching	7	**71 [36 to 92]**
	Numb	16	**75 [51 to 90]**

Bolded text shows rates of understanding ≥70%.

Underlined text shows rates of understanding ≤50%.

### Comparison of spontaneous terms and prompted English terms

The percentage concordance between terms used by participants to spontaneously describe their symptoms in isiZulu and those words selected by them when they were given a list of English terms is shown in Table 6. Participants typically chose equivalent English words from the wordlist to those isiZulu words they used spontaneously. However, participants chose more words to describe their symptoms when they were prompted compared to when they gave a spontaneous description [median (IQR) difference in symptom count: 6 (4–8)].

**Table 6:** Concordance between spontaneously used isiZulu terms and English terms chosen from a list of neuropathic symptom descriptors.

Descriptor	Number of participants who spontaneously used the isiZulu term	Number of participants who chose the prompted English term*	% concordance [95% CI]
***Symptom descriptors***			
Hot	26	24	92 [76 to 98]
Cramping	19	18	95 [75 to 99]
Itching	12	9	75 [47 to 91]
Numbness	12	5	42 [19 to 68]
Cold/freezing	9	8	89 [56 to 98]
Burning	4	2	50 [15 to 85]
Pricking	3	0	0
Knife-like/stabbing	3	2	67 [21 to 99]
Pins-and-needles	1	1	100
Tight	1	1	100
***Symptom elicitors***			
Caused by pressure	11	7	64 [35 to 85]
Caused by cold	2	2	100
Caused by heat	1	0	0

* Only participants who had used the word in their spontaneous descriptions were included in this analysis.

## Discussion

We investigated the terms used spontaneously by native speakers of a commonly spoken Bantu language, isiZulu, to describe their neuropathy symptoms. We also investigated the patients’ knowledge and understanding of English terms typically used in neuropathic pain screening tools and symptom inventories. The five most frequently used spontaneous isiZulu descriptors included equivalents of the English terms: “hot/burning”, “cramping”, “painful/sore/aching”, “itching”, and “numb”. There was a high level of concordance between these spontaneously expressed terms and the terms selected from the English word list, which indicates significant overlap in the nature of the descriptors commonly used across the two languages. Also, knowledge and understanding of the English terms that were equivalent to the five most commonly used isiZulu descriptors was good, except for the English term “numb”. Almost two-thirds of patients were not familiar with the English word “numb”, despite over 20% of patients spontaneously describing a lack of feeling in their feet and legs in isiZulu. Terms describing the spatial quality of the pain (“radiating”, “jumping, “shooting”) were seldom used to spontaneously describe the symptoms in isiZulu or when prompted with English terms, and understanding of these English terms was poor.

A potential source of error in this study was the reliance on accurate translation of the isiZulu symptom descriptions. However, by generating a consensus translation based on the outputs of two independent translators, who were native isiZulu speakers that were fluent in English, we believe that we achieved accurate translations of the isiZulu component of the interview. Another potential limitation was the use of a convenience sample of patients with symptomatic HIV-associated sensory neuropathy instead of a cohort of patients with a variety of different neuropathic conditions. However, there is no clear indication that the symptomatology of neuropathic pain is pathognomonic for the precipitating event (e.g., HIV, diabetes, trauma) [2], [20]–[22], and so we believe that the symptomatology we assessed in our patients will be broadly applicable to other causes of symptomatic peripheral neuropathy.

The most common spontaneously expressed symptom in isiZulu was “hot/burning”. The terms “hot” and “burning” commonly are found on neuropathic pain screening tools, and offer good discriminative properties when distinguishing neuropathic from non-neuropathic pain [1], [2], [23]. Indeed, “hot” or “burning” were chosen as key neuropathic pain descriptors by international experts during a recent Delphi survey to establish a consensus case-definition of neuropathic pain for epidemiological research [24]. Our study participants also frequently used isiZulu terms describing “itching”, “cold pain”, “loss of feeling”, and “pricking/pins-and-needles” sensations to describe their symptoms. All these symptoms are used in at least one commonly used and validated neuropathic pain screening tool [1]. Thus, despite cultural and linguistic differences between isiZulu and Indo-European languages, much of the core symptomatology of neuropathic pain is apparently highly conserved, indicating that cross-cultural translation of existing non-African language screening tools into a Bantu language may be practical. Care would be needed, however, when providing short and meaningful translations for punctate mechanical stimuli (e.g., “pricking”, “pins-and-needles”), where contextual use of a single term was important in our cohort.

IsiZulu equivalents of the terms “tingling”, “electric shocks” and “shooting” were not used spontaneously by our patients, but these terms form part of the symptomatology assessed by several screening tools [1], [2] and were identified in a recent Delphi survey as being important to a case definition of neuropathic pain [24]. The absence of use of these three symptoms in the spontaneous isiZulu descriptions may indicate that these sensations were not experienced by our patients or that these terms do not form part of our patients’ pain vocabulary. Yet, “tingling”, “electric shocks” and “shooting” were chosen from the English list, which indicates that these symptoms may have been experienced but were possibly not used spontaneously because they were not salient features of the pain. Interpretation of the data obtained from the English list does however need to be done cautiously. When we prompted our study participants with the English descriptors “tingling”, “electric shocks”, “pricking”, and “shooting” these terms were not known by many participants. And, when patients did identify a particular English term, only one-third of patients had a true understanding of the terms “tingling” and “shooting”, and more than a third did not have a true understanding of the terms “pricking” and “electric-like”. It also is difficult to determine whether our patients had a true understanding of jargon terms such as “pins-and-needles”. For example, isiZulu descriptions of the English term “pins-and-needles” sometimes included terms whose direct translation is “needles” (“inalithi” or “izinalithi”), and therefore it is difficult to judge if patients truly understood the essence of a pins-and-needle-like sensation even though they may have understood the literal meaning of the English terms “pins” and “needles”.

Our data illustrate a clear gap in comprehension between some terms that are routinely assessed in English neuropathic pain screening tools and what non-native speakers of the language may be familiar with. This comprehension gap could lead to significant under-reporting of neuropathic pain if English screening tools are used in our patient population. The problem of English proficiency when using English versions of screening tools is highlighted by the case of the symptom “numbness”, which is assessed in all major neuropathic pain screening tools except the LANSS [1]. Over 20% of patients spontaneously described a lack of feeling in their feet, but only about 20% of patients interviewed knew and understood the English term “numb”. This discrepancy led to a low level of concordance between the spontaneous and prompted use of this symptom.

“Cramping” was the second-most used term when participants spontaneously described their neuropathy symptoms, and was the most commonly selected term when they were prompted with the list of English terms. The English term was almost universally understood by patients. Indeed, in addition to using the isiZulu term “amajaqamba,” to describe cramp-like pain, many participants used isiZulu derivatives of the English term, such as “ama-cramps” or “namacramps’’, to describe their pain. Our finding that neuropathic pain may be cramping in quality is consistent with the results obtained by Dubuisson and Melzack [25] and Masson and colleagues [26], who found that “cramping” was one of the common terms used to describe the symptoms of neuropathic pain in patients with phantom limb pain and painful diabetic neuropathy. Bouhassira and colleagues [27] identified that almost two-thirds of patients with neuropathic pain, of various aetiologies, used the constrictive terms “squeezing” and “pressure” to describe their pain, but neither term was discriminatory between neuropathic and nociceptive pain [18]. Thus, although the term “cramping” was used frequently by our cohort, it remains to be determined whether the term is discriminatory between neuropathic and nociceptive pain in our patient group. Nevertheless, clinicians should take note of the frequent use of the term “cramping” by patients with HIV-SN to describe their pain, and should render further investigation for possible peripheral neuropathy when the term is used because this sensation may be the most salient feature of the pain to the patient.

In summary, we provide the first evidence of robust similarity in the symptomatology of neuropathic pain between a major Bantu language, isiZulu, and non-African languages. We determined this similarity directly by asking patients to spontaneously describe their symptomatology in their native language rather than inferring similarity indirectly through testing the sensitivity and specificity of translated neuropathic pain screening tools. Moreover, we demonstrate high variability in the knowledge and understanding of English neuropathic terminology by moderately educated native isiZulu speakers who spoke English as second language. Based on our findings we believe that translation of existing questionnaires into isiZulu, and possibly other related Bantu languages, is a legitimate pursuit. But, the use of these tools in their English format, as has been recommended [28], has limitations.
